# Discerning Localized Thermal Heating from Mechanical Strain Using an Embedded Distributed Optical Fiber Sensor Network

**DOI:** 10.3390/s20092583

**Published:** 2020-05-01

**Authors:** R. Brian Jenkins, Peter Joyce, Adam Kong, Charles Nelson

**Affiliations:** 1 Department of Electrical and Computer Engineering, US Naval Academy, 105 Maryland Ave., Annapolis, MD 21402, USA; adamk_97@yahoo.com (A.K.); nelson@usna.edu (C.N.); 2 Department of Mechanical Engineering, US Naval Academy, 590 Holloway Rd., Annapolis, MD 21402, USA; pjoyce@usna.edu

**Keywords:** distributed optical fiber sensors, temperature sensors, polymer matrix composites, high energy radiation, strain sensors, structural health monitoring, smart structures, strain compensation

## Abstract

Prior research has demonstrated that distributed optical fiber sensors (DOFS) based on Rayleigh scattering can be embedded in carbon fiber/epoxy composite structures to rapidly detect temperature changes approaching 1000 °C, such as would be experienced during a high energy laser strike. However, composite structures often experience mechanical strains that are also detected during DOFS interrogation. Hence, the combined temperature and strain response in the composite can interfere with rapid detection and measurement of a localized thermal impulse. In this research, initial testing has demonstrated the simultaneous response of the DOFS to both temperature and strain. An embedded DOFS network was designed and used to isolate and measure a localized thermal response of a carbon fiber/epoxy composite to a low energy laser strike under cyclic bending strain. The sensor interrogation scheme uses a simple signal processing technique to enhance the thermal response, while mitigating the strain response due to bending. While our ultimate goal is rapid detection of directed energy on the surface of the composite, the technique could be generalized to structural health monitoring of temperature sensitive components or smart structures.

## 1. Introduction

Optical fiber sensors provide several advantages for detecting temperature or strain in polymer matrix composites [1,2,3]. They are relatively noninvasive and lightweight. Though an optical fiber is fragile, it is sufficiently flexible to embed in composite structures, and optical fiber sensors respond to changes in strain or temperature quickly and with high sensitivity [4], even at very high temperatures (approaching 1000 °C) [5,6,7].

Distributed optical fiber sensors (DOFS), specifically DOFS based on Rayleigh scattering, can detect temperature or strain with high spatial resolution [8]. This makes them attractive for use in applications requiring the detection of a localized perturbation (in temperature or strain), such as fire or impact damage in composites. Examples include detection of fire, high energy radiation, and the resulting damage on aerospace or mechanical structures [9,10], in which localized, high temperature gradients must be rapidly detected to protect the structure [11]. However, the detection time required to rapidly measure temperature variations when using a fiber optic sensor can be limited by either the thermal response time of the host material or strains that may be present in the structure at any given instant and that can mask the thermal response. Various fiber sensors that can be used in composites to perform structural health monitoring, along with their comparative advantages and disadvantages, are provided in [11].

Past research has shown that embedded fiber Bragg gratings (FBGs) can be used to accurately measure the temperature and location of high energy laser (HEL) strikes on composites [12]. However, as point sensors, numerous FBGs must be dispersed throughout the composite to accurately identify the location of a strike. In contrast, DOFS are distributed sensors with numerous sampling points along the entire length of the fiber that can be spatially resolved using swept wavelength interferometry, with greater likelihood of detecting and locating a strike. The focus of this effort was to use localized heating in the presence of applied mechanical strain to test a concept for strain cancellation in a properly configured DOFS network [13] that would mitigate the effect of bending strain on the rapid detection of a HEL strike.

In previous research [14], DOFS in bare fiber were calibrated to higher temperatures than had been previously published. Specifically, a single 1-m long DOFS was embedded within a polymer matrix composite and subsequently assaulted with a high energy laser (HEL). Temperatures over 900 °C resulted during the laser strike, and the speed of the sensor response was determined. In general, the laser strike was detected in less than 1 sec, with the fastest detection time as low as 42 ms, dependent on the position of the strike relative to the sensor. In the additional testing described in this paper, a carbon fiber reinforced polymer (CFRP) specimen is tested with applied cyclic bending strain. Since strain in the specimen can obscure the response due to temperature, resulting in slower detection, a signal processing technique is described that cancels the bending strain measured in the specimen, while also amplifying the thermal response. Since the goal is rapid detection of a temperature spike during an HEL strike—and well before temperatures become excessive—the technique is first tested using low energy radiation from an optical fiber pigtailed laser diode, to emulate the early stage of a HEL strike. 

This paper first briefly describes the operation of a distributed optical fiber sensor based on Rayleigh scattering. The procedures used to embed the DOFS and the sensor network configuration in the composite are then explained. The test configuration for a composite beam under cyclic loading with and without low energy laser radiation is described. Predictions are made if mechanical strain or heat is applied. Results are presented first with only strain applied to the composite to achieve a baseline test of strain cancellation, then using only a laser diode to generate localized heating to various temperature levels. Finally, test results are described when both strain and heating are present in the composite specimen, to demonstrate how the temperature response can be enhanced as the strain response is canceled.

## 2. Theory and Background

Distributed sensing based on Rayleigh backscattering can be achieved using standard optical fiber made of silica. Small variations in the density of the glass occur during the initial draw of the fiber, so that each fiber is uniquely characterized by an index of refraction that varies randomly along the length of the optical fiber [15,16]. The index of refraction is sensitive to strain or temperature fluctuations, due to the photoelastic and thermooptic effects in silica, respectively [11,12,13]. Optical frequency domain reflectometry (OFDR) can be used to detect changes in the index of refraction, so that each optical fiber can be used as a distributed sensor to measure changes in temperature or strain that temporarily (or permanently) alter the back reflected signal from its initial (i.e., reference) state. An interrogator system reads the backscattered amplitudes gathered during the tests, and after appropriate signal processing, the cross correlation of the received signals with the reference state of the sensor yields a frequency shift that (when properly calibrated) corresponds to changes in strain or temperature. For the Luna Innovations HD-FOS sensors used here, the temperature and strain were measured with a spatial resolution of ~1 mm along the full length of a ~1 m long sensor. 

At low temperatures, such as those used in these tests, the relationship between the frequency shift measured by the Luna Innovations Optical Distributed Sensor Interrogator (ODiSI-B) and the temperature is linear. Equation (1), provided by Luna for this particular sensor, is valid for temperatures below approximately 250 °C, where the proportionality coefficient relating the temperature shift to frequency has units of °C/GHz:

(1) ΔT=−0.6800f.

For larger temperature shifts, such as those that occur during a HEL strike, the sensitivity relationship is nonlinear, described using a higher degree polynomial [14]. The relationship between the frequency shift and strain can also be defined using a sensitivity polynomial. For the Luna Innovations ODiSI-B interrogator and the sensor used in these tests, the Luna-provided relationship is

(2) $$\varepsilon (f)=-\left(a\;f^{2}+b\;f\right)=-\left(5.6193\times 10^{-5}f^{2}+6.6888f\right),$$

where the coefficients *a* and *b* relating the strain ε to the frequency shift *f* are given in με/GHz^2^ and με/GHz, respectively.

Additional specifications provided by the manufacturer for the sensors used in these experiments include: gage length, 1.3 mm; gage pitch, 0.65 mm; strain measurement range and resolution, 10000 ± 1 με; maximum temperature range and resolution, 220 ± 0.1 °C; data acquisition rate, 23.8 Hz. For the strain specifications in these sensors, the second order term in (2) has a minimal effect and the frequency relationship to strain is approximately linear.

## 3. Specimen Preparation and Predicted Strain Response

For the current tests, in order to embed the DOFS into CFRP, a 30 cm × 28 cm swatch of plain weave carbon fiber fabric was first wetted out with a slow curing epoxy resin on a smooth granite surface. To fabricate a 6-ply CFRP specimen, the excess resin was scraped off of the fabric and the saturated carbon fabric was cut into six 5 cm × 28 cm rectangles. Then, one of the six plies was laid on a clean area of the granite table. The DOFS was placed along the centerline of the ply lengthwise and taped down on both sides, so that the optical fiber was straight and taut. Two additional plies of the prepared CFRP specimen were then placed on top of the first ply, and a second segment of the DOFS was looped back and taped in place the same way as before—that is, lined up with the optical fiber beneath it. Two additional plies of the carbon fiber specimen were subsequently placed on top and a third segment of the DOFS was taped in place the same way. Finally, the last ply was positioned on top of the composite specimen, and a thin strand of Kevlar was set along the top of the composite, directly above where the DOFS was embedded in order to mark the location of the DOFS inside the composite. Note, when embedding the DOFS, a loop of exposed optical fiber was present on each end where the sensor exited and then entered back into the composite. Since the optical fiber is delicate, care was taken to create a loop of exposed fiber that was sufficient to ensure that it was no smaller than the minimum bend radius (of ~1 cm) of the fiber to prevent breakage.

The composite was then vacuum bagged at approximately one atmosphere of pressure. A single layer of breather cloth was used in the layup together with a layer of peel ply, to allow the vacuum bag to breathe and to soak up excess resin. A vacuum bag quick disconnect connector was placed on top of the breather cloth, where the vacuum hose then exited the vacuum bag. The composite was then sealed in the vacuum bag and left to cure at room temperature for 24 h. After 24 h, the vacuum pump was turned off and the vacuum bag was removed. The breather cloth was removed, exposing the composite. Using a razor blade, the 6-ply composite with the embedded DOFS was carefully removed from the granite table. 

**Figure 1:**
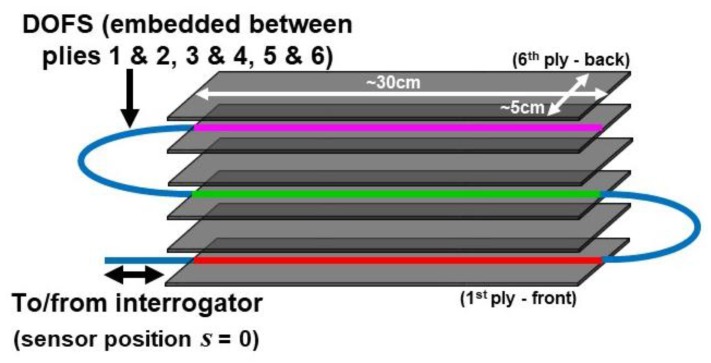
Schematic of beam illustrating distributed optical fiber sensors (DOFS) network between carbon fiber reinforced polymer (CFRP) plies.

**Figure 2:**
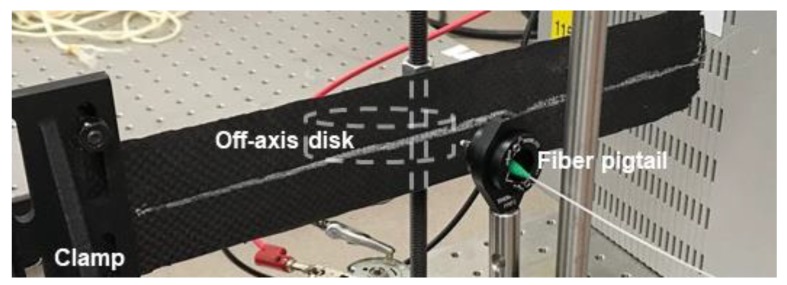
Photo of 6-ply CFRP composite with embedded DOFS. The beam is clamped on the left side in the figure; the off-axis disc to apply strain is hidden behind the specimen (mounted on a vertical rod); the optical fiber pigtail from the laser diode is in front of the specimen.

Figure 1 shows a schematic of the DOFS network in the six-ply CFRP beam and Figure 2 shows a photograph of the beam. As just described, the ~1-m long DOFS was embedded between the top two plies, the middle two plies, and the bottom two plies of the CFRP specimen. These segments are highlighted in color for future reference.

To apply bending strain, the specimen was clamped on one end, as illustrated in Figure 2, and an off-axis disc was placed against the back of the specimen and driven by a DC motor. The disc rotation and distance *L* from the clamp could be adjusted to vary the magnitude of the flexural strain. After measuring the strain response, additional tests were performed with a 975-nm optical fiber-coupled laser diode to irradiate (and heat) various locations on the specimen. 

**Figure 3:**
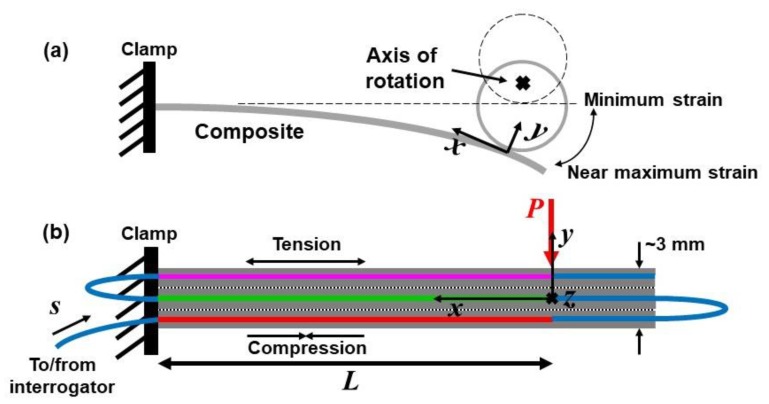
(**a**) Schematic of clamp, off-axis disc and composite specimen. The specimen is clamped on the left side; the disc rotates to apply varying loads to the right side on the back of the specimen. (**b**) Beam diagram showing the clamp, the applied load *P* and the relative location of the DOFS in the six plies of the CFRP specimen. The different segments of the DOFS where strain is measured are highlighted with different colors. The front of the specimen (red) experiences compression; the back (magenta) experiences tension. The green segment is on the neutral axis; the blue segments experience no strain.

A schematic and a beam diagram for the full experimental setup are shown in Figure 3. Figure 3a illustrates the range of beam displacement due to rotation of the off-axis disc, and defines the *x-y* coordinate system with respect to the mechanical load. The corresponding beam diagram in Figure 3b depicts the relative locations of the DOFS through the thickness of the composite (color-coded consistent with the schematic in Figure 1). The thickness of the 6-ply specimen (in the y-direction), including the embedded fiber sensor, is approximately 3 mm. The profile of the strain ε=Δℓ/ℓ (typically in με) that results is given by

(3) $$\varepsilon (x,y)=\frac{Pxy}{EI},$$

where *P* is the applied load, *x* and *y* represent the position along the beam away from the load and the position off the neutral axis, respectively, *E* is Young’s modulus, and *I* is the moment of inertia. Hence, Segments 1 and 3 of the DOFS labeled in red and magenta should experience compression and tension, respectively, for the load applied as shown. Segment 2 of the fiber labeled in green is on the neutral axis (ideally at *y* = 0), and the blue segments (the unloaded fiber ends exiting and entering the composite) of the DOFS are not loaded. Position *s* = 0 in the sensor is defined near the interrogator, and the total length of the sensor is almost 1.2 m.

**Figure 4:**
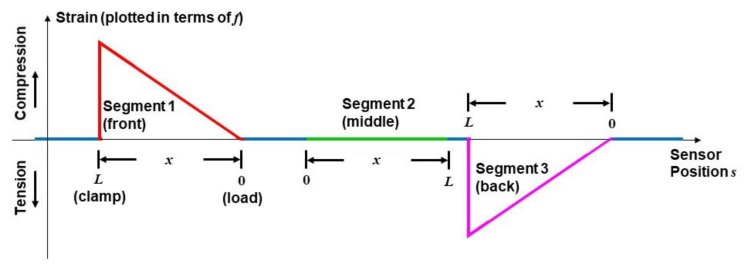
Predicted strain measured by the embedded DOFS (plotted in terms of GHz) for the specimen in Figure 1, as functions of sensor position *s* or beam position *x*.

Based on Equations (2) and (3) for the sensor network configuration in Figure 1, the predicted strain profile detected by the DOFS under cyclic loading at maximum displacement is as shown in Figure 4. Compressive or tensile strain at each sensor position *s* is plotted in terms of frequency shift *f*(*s*), consistent with how the interrogator will display the response, measuring a positive frequency shift under compression when ε < 0 (and a negative frequency shift under tension when ε > 0). The colors in Figure 4 correspond to the highlighted segments used for the DOFS in Figure 1 and Figure 3. 

**Figure 5:**
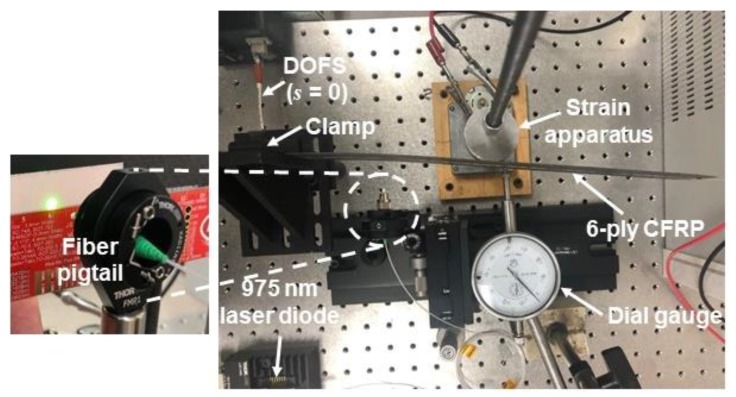
Experimental setup used to apply strain and localized heating to the CFRP specimen. The photo of the end of the fiber pigtail shows measurement of the spot size using a ruler against an IR viewing card.

**Figure 6:**
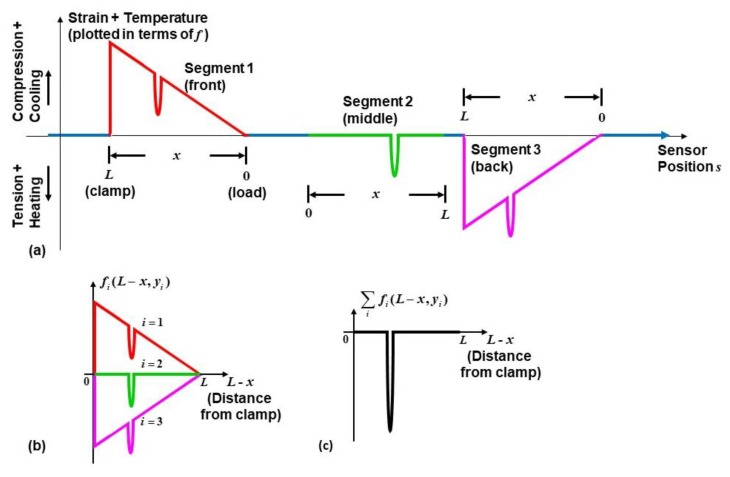
(**a**) Predicted response due to both strain and temperature changes measured by the embedded DOFS (plotted in GHz) for the specimen in Figure 1, using the test setup in Figure 5. (**b**) Frequency shift in each segment as a function of the beam position relative to the clamp, L−x, and (**c**) the aggregate response $\sum_{i}f_{i}$ that isolates the thermal response.

To generate heat in the CFRP, a relatively high power (600 mW maximum) laser diode at 975 nm was used. The experimental setup is shown in Figure 5. The laser energy was emitted from the fiber optic pigtail on the laser diode. The end of the pigtail was mounted on translation stages with micrometer adjustments to precisely set the location of the laser relative to the specimen. When the tip of the ferrule at the end of the fiber pigtail was 1.4 cm from the specimen, the spot size of the emitted laser light on the specimen was ~4 mm in diameter, as shown on the IR (infrared) viewing card in the inset photo in Figure 5. When the laser light is incident on the specimen, the applied energy causes a localized temperature change in the interrogator response curve. The resultant frequency shift measured by the interrogator can include contributions from both strain and localized heating, as depicted in Figure 6a. Again, the frequency relationships in Equations (1) or (2) are opposite in sign to changes in temperature or strain, respectively, so heating is indicated by a downshift in frequency. 

In order to isolate the thermal response from the strain, first, consider a frequency shift in each segment of the DOFS caused by both strain and changing temperature, as shown in Figure 6a, defined in the x-y reference frame of the beam,

(4) $$f_{i}(x,y_{i})=\varepsilon_{i}^{-1}(x,y_{i})+\Delta T_{i}^{-1}(x,y_{i}),$$

where $y_{i}=[-y,0,y]$ for Segment *i* = 1, 2, or 3. Here, $\Delta T_{i}^{-1}(x,y_{i})$ and $\varepsilon_{i}^{-1}(x,y_{i})$ are the inverse functions of Equations (1) and (2), respectively, at all positions between the load and the clamp, where strain is present under steady state conditions and any temperature response normally occurs as a rapidly changing transient (or impulse) at a localized position on the specimen. Based on Equation (2), the component of the frequency shift (in GHz) resulting from strain in the composite is

(5) $$\varepsilon_{i}^{-1}(x,y_{i})=\frac{-b+\sqrt{b^{2}-4a\varepsilon_{i}(x,y_{i})}}{2a},$$

with $a=5.6193\times 10^{-5}\;$με/GHz^2^ and b=6.6888 με/GHz. However, for a maximum specified strain of ε=10,000 με in the Luna sensor, the approximation $\varepsilon_{i}^{-1}(x,y_{i})\approx -\varepsilon_{i}(x,y_{i})/b$ holds, therefore the component of the frequency shift $f_{i}(x,y)$ due to strain is linearly related to $\varepsilon_{i}(x,y_{i})$ for all measurable values of strain. Furthermore, assuming sensor Segments 1 and 3 locations are symmetric about the neutral axis and that Segment 2 lies on the neutral axis, if $f_{i}(x,y)$ is measured independently in each segment and added, the contributions to $\sum_{i}f_{i}(x,y)$ due to strain will, in theory, cancel completely since $\varepsilon_{1}^{-1}(x,-y)+\varepsilon_{2}^{-1}(x,0)+\varepsilon_{3}^{-1}(x,y)\approx -\sum_{i}\varepsilon_{i}(x,y_{i})/b\approx 0.$ Hence, the aggregate response in GHz will represent an amplified version of the temperature shift due to the incident laser light, isolated from the strain, as in

(6) $$\sum_{i=1}^{3}f_{i}(x,y_{i})\approx 3\Delta T^{-1}(x)=-\frac{3\Delta T}{0.6800}.$$

In Equation (6), the measured temperature shift in each segment is assumed for now to be approximately the same through the thickness of the specimen; in other words, ΔT is independent of *y*. Figure 6b,c illustrate the isolation technique graphically, with each segment response $f_{i}(L-x,y_{i})$ and the aggregate response $\sum_{i}f_{i}(L-x,y_{i})$ plotted as a function of the distance from the clamp, L−x.

Further adaptations of the DOFS network can be explored; as an example, increasing the number of DOFS segments to five or seven could provide enhanced amplification of the thermal response, while still eliminating the mechanical response. The signal processing technique implemented in Equation (6) is analogous to the use a full Wheatstone bridge to amplify the sensitivity of strain gage response for stress analysis [17]. This technique could also be applied in embedded networks of other optical sensors that detect both strain and temperature, such as FBGs, or perhaps when using other novel sensing materials that we have considered [18,19,20,21]. The next section demonstrates the experimental implementation of the isolation technique.

## 4. Demonstration of Strain Isolation and Thermal Response

### 4.1. Strain Cancellation 

**Figure 7:**
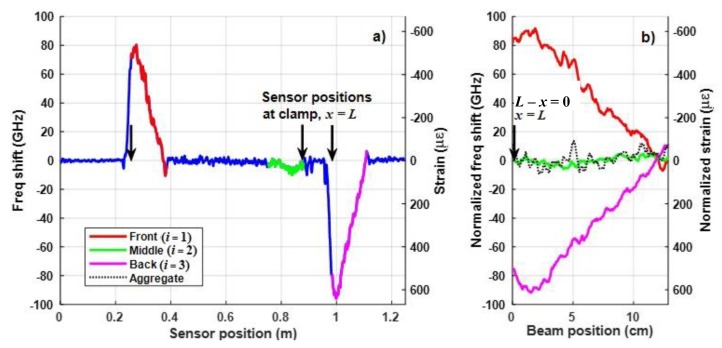
(**a**) Time-averaged frequency shift ${\langle f(s,t)\rangle }_{t}$ (left axis) and strain ${\langle \varepsilon (s,t)\rangle }_{t}$ (right axis) vs. sensor position *s* for the maximum strain condition, using the strain apparatus in Figure 2. (**b**) Based on Equation (7) for Segments *i* = 1-3, corresponding plots of the normalized frequency shift $\left(f_{iss}(L-x,y_{i})-{\bar{f}}_{2ss}\right)S_{i}$ and the aggregate shift $f_{agg-ss}(L-x)$ (black dotted line) vs. the beam position *L – x* (relative to the clamp). The normalized strain is shown on the right axis.

During initial tests, only a mechanical load (with no heating) was applied to the composite specimen in order to verify the expected strain profiles in Figure 4. While applying bending strain as shown in Figure 2 and Figure 3, numerous measurements were taken to demonstrate repeatability. Both static and dynamic (cyclical) strain were applied at various load levels, i.e., the steady state condition without directed energy present. Figure 7a shows a plot of the time-averaged values of the measured frequency shift ${\langle f(s,t)\rangle }_{t}$ versus sensor position *s* for maximum strain under static loading (see Figure 3a). The mechanical load was applied at a position near the middle of the specimen, approximately 12 cm from the clamp. The plot is highlighted using the same color coding of Segments *i* = 1–3 of the DOFS, as defined in Figure 4 and Figure 6 (labeled as Front, Middle and Back, respectively). The right axis of the plot in Figure 7a shows the corresponding numerical values of the time-averaged steady state strain ${\langle \varepsilon (s,t)\rangle }_{t}\approx -b\cdot {\langle f(s,t)\rangle }_{t}$. Within each segment (in terms of beam position *x*), the measured frequency shifts plotted in Figure 7a are mathematically described as $f_{iss}(x,y_{i})={\langle \varepsilon_{i}^{-1}(x,y_{i},t)\rangle }_{t},$ where $f_{iss}(x,y_{i})$ in GHz corresponds to the time-averaged strain response in steady state, i.e., before directed laser energy is applied. The time-average was applied to mitigate noise fluctuations over the full time span of the sensor interrogation, typically lasting several seconds. In practical real-time application, the strain occurring in steady state would likely be measured as a running average. 

As Figure 7a illustrates, the strain response of the CFRP beam is generally consistent with the predictions in Equation (3) and Figure 4. The DOFS segment near the front face of the specimen (Segment 1, red curve) experiences compression where *f* > 0, and with strain that varies from a peak value near the clamp linearly to a minimum near the applied load. The strain detected near the back face (Segment 3, magenta curve) also varies linearly, but the rear face is in tension with *f* < 0. In contrast, the strain measured in the middle segment of the DOFS (Segment 2, green curve) near the neutral axis is relatively small, as expected, compared to the strains measured near the outer faces of the CFRP. 

However, Figure 7a also indicates that there are differences in the relative magnitudes of the measured strain near each face. Segments 1 and 3 of the DOFS were designed to be equidistant from the neutral axis, so that they would experience equal but opposite bending strain, but the placement of the DOFS was slightly asymmetric about the neutral axis of the beam. Consequently, $\varepsilon_{1}^{-1}(x,y_{1})\neq -\varepsilon_{3}^{-1}(x,y_{3})$ in the red and magenta plots, because $\left|y_{1}\right|\neq y_{3}$ at sensor positions between the clamp and the load. Furthermore, the average strain on the neutral axis in Segment 2 (in green), though small, is also not equal to zero over the length of the sensor. The mean frequency shift on the neutral axis (computed over length *L* ≈ 12 cm) was approximately -4.0 GHz representing an average strain of approximately 27 με based on Equation (2). As would be expected, deformations in the specimen (e.g., due to variations in the thickness of each ply, molding artifacts during composite preparation or the amount of resin applied during fabrication) cause uncertainty in the precise location of the neutral axis through the thickness of the structure. Hence, the middle segment of the DOFS in between the third and fourth plies is not always coincident with the neutral axis.

Consequently, to empirically optimize the strain cancellation technique defined in Equation (6), the responses in each segment must be normalized. First, the mean steady state value of the frequency shift ${\bar{f}}_{2ss}={\langle f_{2ss}(x,y_{2})\rangle }_{x}$ in Segment 2 is removed (subtracted) from the overall response along the full length of the sensor fiber, which shifts the data in Segments 1 and 3 about a neutral axis, redefined as Segment 2. Furthermore, in order to account for asymmetry between the outer plies in Segments 1 and 3 that might occur during specimen preparation, the strain response measured near either of the outer faces must also be rescaled before computing the aggregate response from all three DOFS segments. Mathematically, after normalizing Equation (6), the optimized aggregate response becomes:

(7) $$f_{agg-ss}(x)=\sum_{i=1}^{3}\left(f_{iss}(x,y_{i})-{\bar{f}}_{2ss}\right)S_{i},$$

where the rescaling factor has been arbitrarily applied to Segment 1, as in

(8) $$S_{i}=\left[S_{1},\;S_{2},\;S_{3}\right]=\left[\frac{\max\left|f_{3ss}(x,y_{3})-{\bar{f}}_{2ss}\right|}{\max\left|f_{1ss}(x,y_{1})-{\bar{f}}_{2ss}\right|},\;1,\;1\right].$$

The normalized frequency responses $\left(f_{iss}(L-x,y_{i})-{\bar{f}}_{2ss}\right)S_{i}$ in Equation (7), based on the data in Figure 7a, are plotted in Figure 7b, as functions of the beam position (defined as the distance from the clamp) *L – x*, where red, green and magenta lines are used for Segments *i* = 1, 2, or 3, respectively. Specifically, the results in Figure 7a are characterized by ${\bar{f}}_{2ss}\approx -4.0\;\mathrm{GHz},$ with maximum and minimum frequency shifts of $f_{1ss}(x\approx L,y_{1})=80\;\mathrm{GHz}$ and $f_{3ss}(x\approx L,y_{3})=-95\;\mathrm{GHz}$ that correspond approximately to compressive strain of –535 and tensile strain of 635 με. For these values, the resulting scale factor applied to the data in Segment 1 is $S_{1}=91\;\mathrm{GHz}/84\;\mathrm{GHz}=1.08$ so in Equation (8) $\left[S_{1},S_{2},S_{3}\right]=[1.08,\;1,\;1]$. This suggests that sensor Segment 3 is ~8% farther from the neutral axis than Segment 1. As implied by Figure 6b, if the isolation technique works properly, the aggregate shift $f_{agg-ss}(x)$, calculated using Equation (7) and shown by the black dotted line in Figure 7b, should ideally be 0 Hz once strain is canceled and there is no heating. The mean frequency shift in the aggregate response in Figure 7b is 1.13 GHz, corresponding to an average canceled strain of −7.6 με using (2). This aggregate (combined) mean is approximately 28% of the original unscaled mean frequency shift of −4.0 GHz measured on the neutral axis, so the isolation technique was relatively successful at removing the strain response. The standard deviation in the aggregate response $f_{agg-ss}(x)$ is just over 5 GHz. 

### 4.2. Temperature Testing

**Figure 8:**
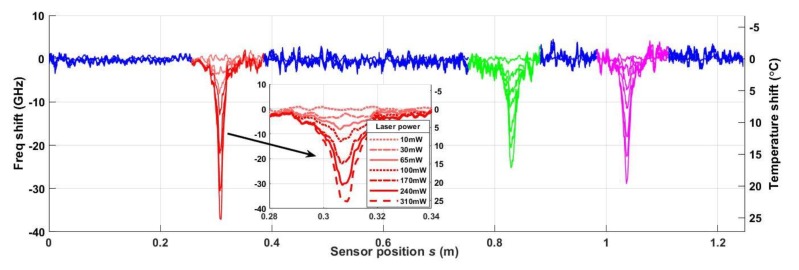
Time-averaged frequency shift ${\langle f(s,t)\rangle }_{t}$ (left axis) and corresponding temperature shifts ${\langle \Delta T(s,t)\rangle }_{t}$ (right axis) as function of sensor position s at seven optical power levels in the 975 nm laser diode.

To obtain a baseline thermal response in the specimen when heat is present, bending strain was removed, and optical radiation from the 975 nm laser diode was applied as shown in Figure 5. Figure 8 shows the resulting time-averaged frequency shifts ${\langle f(s,t)\rangle }_{t}$ as a function of the sensor position *s*, and the corresponding temperature shifts ${\langle \Delta T(s,t)\rangle }_{t}$ (right axis), calculated using Equation (1), at seven different optical power settings on the laser diode. The inset is a zoomed-in view showing the increasing frequency (and temperature) shifts for increasing power levels in Segment 1 (in red) of the DOFS. Similar relationships between increasing power level and increasing temperature occur in Segments 2 and 3 as well. The time-averaged temperature response is plotted here because the laser power was maintained for the entire time of the measurement. In the real application with a high energy laser, ΔT(s,t) would be a transient response that needs to be detected as rapidly as possible even if strain is present in the steady state. Future tests will use a high energy laser while strain is present to verify that the strain cancellation technique works to isolate a transient high temperature impulse. 

**Figure 9:**
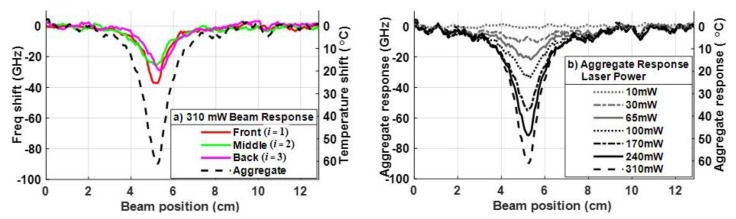
(**a**) Frequency (and temperature) shifts in the front, middle and back segments of the sensor for an optical power of 310 mW (as plotted in Figure 8), along with the aggregate response. (**b**) Amplified aggregate responses based on the measured temperature shifts at all seven optical power levels of the laser diode.

The results in Figure 8 show the temperature responses that should be present, ideally, at each power level if strain were canceled. Using the results from Figure 8 at the 310 mW power level, Figure 9a shows the frequency shift ${\langle f_{i}(x,y_{i},t)\rangle }_{t}={\langle \Delta T_{i}^{-1}(x,y_{i},t)\rangle }_{t}$ vs. beam position *L – x* in each of the Segments *i* = 1–3. The right axis defines the corresponding time-averaged temperature shift ${\langle \Delta T_{i}(x,y_{i},t)\rangle }_{t}$. The dashed line in Figure 9a plots the aggregate (amplified) response $\sum_{i=1}^{3}{\langle f_{i}(x,y_{i},t)\rangle }_{t}$ for the 310 mW laser diode power. No normalization is applied to the aggregate response, since no strain is present. Figure 9b shows the aggregate responses for all seven optical power levels. 

**Figure 10:**
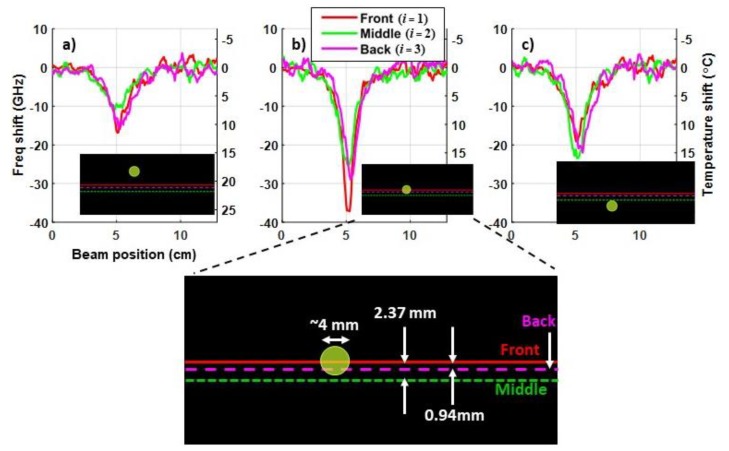
Examples of iterative measurements of frequency and temperature shifts when the ~4 mm laser beam was: (**a**) 5.27 mm above Segment 1 (front) of sensor; (**b**) aligned with Segment 1; and (**c**) 4.73 mm below Segment 1.

Note, Figure 9 shows that the aggregate response is approximately triple the temperature shift in each segment of the sensor. This is expected, as predicted in Equation (6), since the aggregate response is the sum of the actual temperature shifts in the three sensor segments (front, middle and back) in Figure 9a. However, Figure 8 and Figure 9a also show that there were different responses in each segment. While this might suggest that the temperature is non-uniform through the thickness of the CFRP, the vertical translation of the laser demonstrated that the DOFS was imprecisely embedded in the *z* (as well as the *y*) dimension during the layup and curing of the composite. Since the ~4 mm spot size of the laser diode is relatively small, slight differences in the measured temperature in the individual segments of the DOFS can be observed by iteratively adjusting the position of the laser. With this method, Segments 2 and 3 of the sensor were shown to be 2.37 mm and 0.94 mm lower (in the z-dimension of the composite) than Segment 1, respectively, as illustrated in Figure 10. The temperature differences in each segment observed in Figure 8 would be smaller if the laser spot size was larger—as would be common in a typical directed laser energy scenario. Hence, the results in Figure 10 demonstrate that imprecision in aligning the sensor segments in the *z*-dimension is unlikely to impact the strain cancellation technique in Equation (7) when used to detect high energy radiation. 

### 4.3. Simultaneous Application of Strain and Heating

If strain and laser energy are simultaneously applied to the composite, the aggregate response in Equation (7) is modified, such that

(9) $$f_{agg}(x)=\sum_{i=1}^{3}\left(f_{i}(x,y_{i})-{\bar{f}}_{2ss}\right)S_{i},$$

where the frequency shift $f_{i}(x,y_{i})$ defined in Equation (4) is equal to the time-averaged combination of the inverse temperature shift and strain:

(10) $$f_{i}(x,y_{i})={\langle f_{i}(x,y_{i},t)\rangle }_{t}={\langle \Delta T_{i}^{-1}(x,y_{i},t)+\varepsilon_{i}^{-1}(x,y_{i},t)\rangle }_{t}.$$

**Figure 11:**
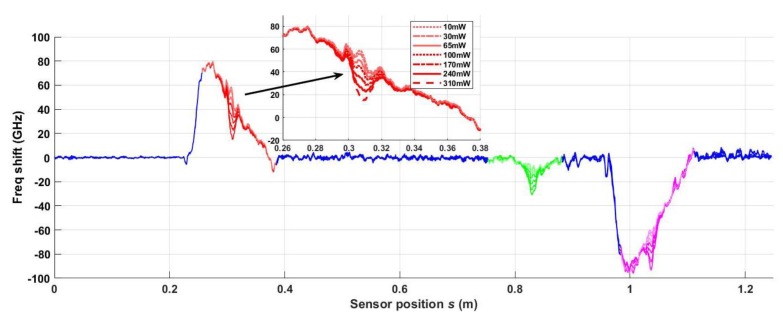
Time-averaged frequency shifts resulting from both strain and the incidence of laser energy at seven optical power levels.

If the strain cancellation technique works properly, the aggregate response due to a temperature shift should be the same with and without strain, so the plots in Figure 9b ideally predict the aggregate responses for each laser power setting, whether strain is present or not. To demonstrate if strain cancellation in Equation (9) succeeds experimentally, the composite was heated, using the same optical power levels in Figure 8 and Figure 9, while applying maximum strain, as shown in Figure 3, with the same strain response as was plotted in Figure 7a. To verify repeatability, the laser was directed at several unique positions along the sensor. The plots in Figure 11 characterize the results well. The strain is linear with distance from the load and with peak values similar to those observed in the strain test described in Figure 7, with ${\bar{f}}_{2ss}\approx -4.0\;\mathrm{GHz}$ and $S_{i}=[1.08,\;1,\;1]$ as before. The resultant frequency shift due to the change in temperature is easy to see—the inset plot in Figure 11 shows finer detail for the seven optical power levels incident on the front segment of the sensor.

**Figure 12:**
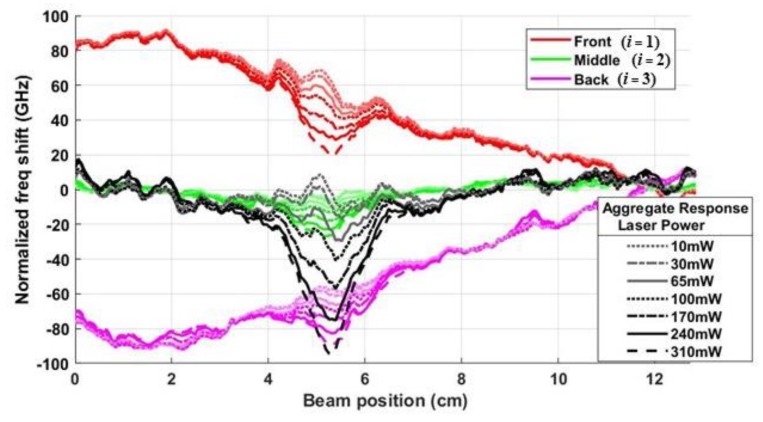
Plots of the normalized responses from Equation (9), corresponding to the combined strain and temperature response in Equation (10), as measured in the front, middle and back Segments of the DOFS (i=1−3). The aggregate response (in black) is the combination of the scaled responses, as defined in Equation (9), for each optical power setting.

Figure 12 shows plots of the normalized frequency responses $\left(f_{i}(L-x,y_{i})-{\bar{f}}_{2ss}\right)S_{i}$ from Equation (9) for the results in Figure 11. The response in each segment of the DOFS is plotted as a function of distance from the clamp *L – x* for all seven optical power settings, where red, green and magenta lines are used for Segments *i* = 1, 2, and 3, as before. The aggregate thermal response $f_{agg}(L-x)$ is the normalized combination of the three responses in (10), as plotted in the black curves in Figure 12. 

**Figure 13:**
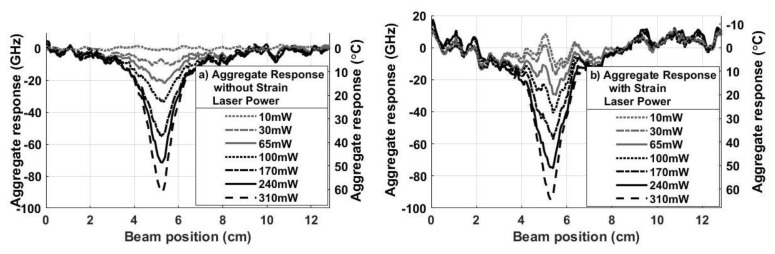
Plots of the aggregate response at each optical power level: (**a**) without strain, and (**b**) with strain.

To more easily compare the response without any strain to the response with bending strain applied to the composite, the aggregate thermal responses in Figure 9b and Figure 12 are plotted alongside each other in Figure 13a,b, respectively. The peak aggregate shift in Figure 13a when no strain was applied is slightly smaller than peak in Figure 13b, as expected, since the data in the aggregate thermal response without strain, plotted in Figure 13a, is only a summation of the individual responses and not normalized as in Equation (9). The aggregate response without applied strain is also somewhat less noisy. However, the noise present in Figure 13b appears to be consistent with the 5 GHz standard deviation in the frequency shift that remained in the aggregate response when only strain was applied to the composite (see Figure 7b). Using Equation (1), a standard deviation of 5 GHz due to strain corresponds to a noise in the acquired temperature shift of 3 °C. This is relatively small, suggesting that a signal processing technique based on Equation (9) is a viable technique to isolate and cancel the strain response if a DOFS is used to rapidly detect localized temperature impulses due to an HEL strike.

## 5. Conclusions

Rapid detection of a thermal impulse due to incident HEL radiation using embedded DOFS in a CFRP composite has been previously demonstrated [12,14]. HEL strikes generate large temperature shifts, however, rapid detection of the strike ideally occurs when the temperature shift is still relatively small. Hence, any strain present in the composite could delay detection of a laser strike. The focus of this effort was to use localized heating in the presence of applied mechanical strain to test a proposed concept for strain cancellation in a properly configured DOFS network, that would theoretically compensate for the effect of bending strain on rapid detection of a HEL strike.

In contrast to prior HEL testing, these experiments required a test configuration that would use relatively low power directed energy and the simultaneous application of bending strain. These experiments resulted in a well-defined strain profile, smaller temperature shifts and no damage to the composite specimen. Testing exposed unanticipated variations in the DOFS alignment and placement that occurred during the embedding process that required additional sophistication in the technique used to cancel the strain response. Additionally, the relatively small spot size of the diode laser used to apply heat made it possible to analyze these unpredicted structural variations with relatively high precision. The modified signal processing algorithm was successfully used to isolate the thermal and strain responses, and the standard deviation in noise associated with the strain response that remained was relatively small. Future experiments will include heating due to high energy laser radiation concurrent with applied mechanical strain, in order to evaluate the effect of bending strain on the ability to quickly detect a laser strike.
